# Clinical characteristics and analysis of risk factors for disease progression of patients with SARS-CoV-2 Omicron variant infection: A retrospective study of 25207 cases in a Fangcang hospital

**DOI:** 10.3389/fcimb.2022.1009894

**Published:** 2022-10-31

**Authors:** Pei Ying-hao, Gu Yuan-yuan, Zhang Hai-dong, Chen Qiu-hua, Gu Xue-ran, Zhou Hai-qi, Jiang Hua

**Affiliations:** ^1^ Department of Intensive Care Unit, Jiangsu Province Hospital of Chinese Medicine, Affiliated Hospital of Nanjing University of Chinese Medicine, Nanjing, Jiangsu, China; ^2^ Department of Medical Affairs, Jiangsu Province Hospital of Chinese Medicine, Affiliated Hospital of Nanjing University of Chinese Medicine, Nanjing, Jiangsu, China

**Keywords:** clinical characteristics, disease progression, COVID-19, SARS-CoV-2 Omicron variant, risk factors

## Abstract

**Objectives:**

To summarize the clinical characteristics of patients infected by SARS-CoV-2 omicron variant and explore the risk factors affecting the progression in a Fangcang hospital, Shanghai, China.

**Methods:**

A total of 25,207 patients were retrospectively enrolled. We described the clinical characteristics and performed univariate and multivariate logistic regression analysis to identify the risk factors for non-severe to severe COVID-19 or death.

**Results:**

According to the outcomes, there were 39 severe patients (including 1 death) and 25,168 non-severe patients enrolled in this study. Among the 25,207 cases, the median age was 45 years (IQR 33-54), and 65% patients were male. Cough (44.5%) and expectoration (38.4%) were the most two common symptoms. Hypertension (10.4%) and diabetes (3.5%) were most two common comorbidities. Most patients (81.1%) were fully vaccinated. The unvaccinated and partially vaccinated patients were 15.1% and 3.9%, respectively. The length of viral shedding time was six days (IQR 4-9) in non-severe patients. Multivariate logistic regression analysis suggested that age (OR=1.062, 95%CI 1.034-1.090, p<0.001), fever (OR=2.603, 95%CI 1.061-6.384, p=0.037), cough (OR=0.276, 95%CI 0.119-0.637, p=0.003), fatigue (OR=4.677, 95%CI 1.976-11.068, p<0.001), taste disorders (OR=14.917, 95%CI 1.884-118.095, p=0.010), and comorbidity (OR=2.134, 95%CI 1.059-4.302, p=0.034) were predictive factors for deterioration of SARS-CoV-2 omicron variant infection.

**Conclusions:**

Non-critical patients have different clinical characteristics from critical patients. Age, fever, cough, fatigue, taste disorders, and comorbidity are predictors for the deterioration of SARS-CoV-2 omicron variant infection.

## Introduction

In December 2019, a new human coronavirus, severe acute respiratory syndrome coronavirus 2 (SARS-CoV-2), was identified and the World Health Organization (WHO) named this infection as coronavirus disease 2019 (COVID-19) (Zhu et al., 2020). The common COVID-19 symptoms are fever, cough, and dyspnea, while less common symptoms are fatigue, headache, anosmia, ageusia, cutaneous manifestation, and gastrointestinal symptoms (Vaira et al., 2020; Geremia et al., 2020; De Vito et al., 2021). Serial SARS-CoV-2 mutants triggered several waves of COVID-19 outbreaks worldwide. To date, WHO has verified six mutants, including the alpha, beta, gamma, delta, and omicron variants, and each later one presents with the stronger transmission ability than the previous (Lv and Lv, 2021). These variants are considered as variants of concerns (VOCs), which possess strong infectiousness or/and intense virulence. According to date from WHO, as of July 11, 2022, the cumulative diagnosed cases of COVID-19 has climbed to over 552 million, and the number of death cases has risen to more than 6.3 million (WHO, 2021).

In November 2021, the Omicron variant (B.1.1.529) of SARS-CoV-2 was first identified in South Africa and Botswana (Venturini et al., 2021). Nowadays, it exceeds other VOCs to become the prevailing SARS-COV-2 strain and sweeps through the globe (Wang et al., 2020). The first case in China was identified on December 9, 2021 (De Vito et al., 2021). BA.1 and BA.2 are major Omicron variant sub-lineages in local COVID-19 outbreaks of China (Chen et al., 2020; Liu et al., 2021; Nishiura et al., 2021). Studies showed that the omicron variant caused a milder viremia than the previous VOCs, which presented as reduced hospital stays and decreased severity and mortality rate (Soy et al., 2020; Ye et al., 2020; Ito et al., 2022). However, the number of viral sequence mutations identified on the omicron variant was higher than that of any other VOCs. Although full course vaccination leads to a serial of antibody productions, partial immune escape should be noticed due to many unknown mutations of the Omicron variant (John et al., 2022). In recent South Africa outbreak wave of the Omicron variant, some cases have already vaccinated with the Pfizer vaccine (Mueller et al., 2022). Since then, several studies indicated that vaccination-induced antibodies could still neutralize the majority of the Omicron mutations, despite some cases showed low affinity and specificity. This is most likely due to the mutation at E484A, which leads to the reduced recognition and neutralization (Kupcewicz et al., 2022). According to clinical data, unvaccinated or partly vaccinated cases have an higher risk of developing severity, which indicates that the vaccines are still effective to prevent severe disease (Elbogen et al., 2022). Chinese anti-epidemic measures have proved that effective treatment of non-critical patients is a powerful tool to reduce the deterioration rate of non-critical cases turning into critical or even death cases. Fangcang belongs to a novel concept hospital of public health for the perspective of treating non-critical COVID-19 patients and controlling the source of infection. It has been proved that Fangcang hospital is beneficial to improve the overburden situation of hospitals and inhibit the wide-bound spread of COVID-19 epidemic (Song et al., 2020; Kupcewicz et al., 2022).

Thus, as the member of Jiangsu province aid to shanghai medical team, we retrospectively analyzed the demographic information and clinical features of 25,207 patients infected with the omicron variant in the Lingang Fangcang hospital during the 2022 Shanghai COVID-19 outbreak. In this study, we intend to ascertain the clinical features of the omicron variant cases and evaluate the risk factors of progression from non-severe to severe COVID-19 or death.

## Methods

### Study design and participants

Clinical Research Ethics Committee of Jiangsu Province Hospital of Chinese Medicine approved this retrospective study (2022NL-159-01) and waived written informed consent since the study was retrospective and was part of a public health outbreak investigation. This study enrolled 25,207 patients diagnosed by RT-PCR test. According to the Shanghai Municipal Health Commission, SARS-CoV-2 viral genomes from 129 patients in this period indicated that the new viral genomes in Shanghai were clustered into the SARS-CoV-2 BA.2.2 sub-lineage, which mean the omicron variant was the primary strain in this Shanghai COVID-19 wave (Zhang et al., 2022). All the patients were hospitalized to Lingang Fangcang Hospital between 5 April and 8 May 2022 in Shanghai, China. Jiangsu medical team was one of the management teams in Lingang Fangcang Hospital.

### Inclusion criteria, exclusion criteria and other critera

The inclusion criteria were as follow: 1) confirmed by COVID-19 nucleic acid RT-PCR test, 2) asymptomatic or mild COVID-19 cases, 3) linked to the recent omicron variant outbreak of COVID-19 originated in Shanghai, 4) adults over 18 years. All enrolled patients were from Lingang Fangcang Hospital, the third largest Fangcang that provided basic medical care, logistics support and living quarters for COVID-19 in Shanghai. Exclusion criteria: 1) cognitive dysfunction, 2) incomplete clinical data, 3) hospitalization in Fangcang less than 24 hours. The criteria for patient admission, transfer and discharge in Lingang Fangcang were presented in **Table 1**. The patients who met the transfer criteria and transferred to the designated hospital were classified as severe cases in this study.

**Table 1:** The criteria for patient admission, transfer and discharge in Lingang Fangcang.

No.	Admission criteria	Transfer criteria	Discharge criteria
1	Asymptomatic or mild symptoms	Respiratory distress, respiratory rate≥30 breathes per min	The body temperature is normal for more than 3 days
2	Have the ability to live and walk independently. The one without any self-care ability should stay with family attendant.	SpO2 ≤ 93% in resting state	Negative test of respiratory virus nucleic acid for 2 consecutive times (sampling time interval is at least 1 day)
3	SpO2>95% and respiratory rate<30 breathes per min in resting state	Cases with chronic diseases deteriorate to decompensation stage	Without oxygen inhalation, SpO2>95%
4.	Cases with chronic diseases, including heart, lung, kidney, brain diseases, were not suffering acute decompensation stage.		Respiratory symptoms improved significantly*
5.	Non-pregnant		
6.	Child <14 years of age needs to stay with a family member		
7.	No history of mental illness, HIV and active tuberculosis		
8.	Cancer patient who is not undergoing chemotherapy		

SpO2 was measured by hand hold pulse oximeter. * This item was one of discharge criteria and a subjective evaluation from patients.

Patients were considered fully vaccinated only if two vaccine doses were received and the time between the second vaccination and the onset of COVID-19 was>14 days. Patients were considered partially vaccinated if only one effective vaccine dose was received. Patients were considered unvaccinated if no vaccine had been received or the first vaccination was given<14 days before the onset of COVID-19. -We have modified. Nasopharyngeal or oropharyngeal swabs were collected every day for first three days of the admission and then every 2 days.

### Treatment and outcomes

The treatment was according to the trial ninth version of COVID-19 guidelines published by the Chinese government. The demography data, basic clinical features and comorbidities were recorded. The primary outcome is recovery and discharge from Fangcang hospital or deterioration and transfer to designated hospital. The viral shedding time (VST) in our study was defined as the duration from the first day of positive RT-PCR result to the first day of two consecutively negative nucleic acid tests (interval more than 24 hours).

### Statistical analysis

IBM SPSS Statistics (version 21.0, IBM, Armonk, NY, USA) was used for data analysis. Continuous data were presented as means and standard deviations (SDs), or medians and interquartile ranges (IQRs). Differences between groups were tested using the student-*t* test, Mann Whitney *U* test, χ^2^ test, or Fisher exact test. A two-sided significance level of 5% was used, and 95% CIs were reported for all analyses. Logistic regression analysis was used to estimate the relative effect of outcome variables by calculating unadjusted odds ratios (ORs). The variables that significantly different (P<0.05) between severe and non-severe groups were selected for univariate analysis. In the univariate analysis, variables of p-value less than 0.05 were selected for further multivariate modeling analysis. The significance level was set at 0.05.

## Results

### Baseline demographic and clinical characteristics

**Figure 1:**
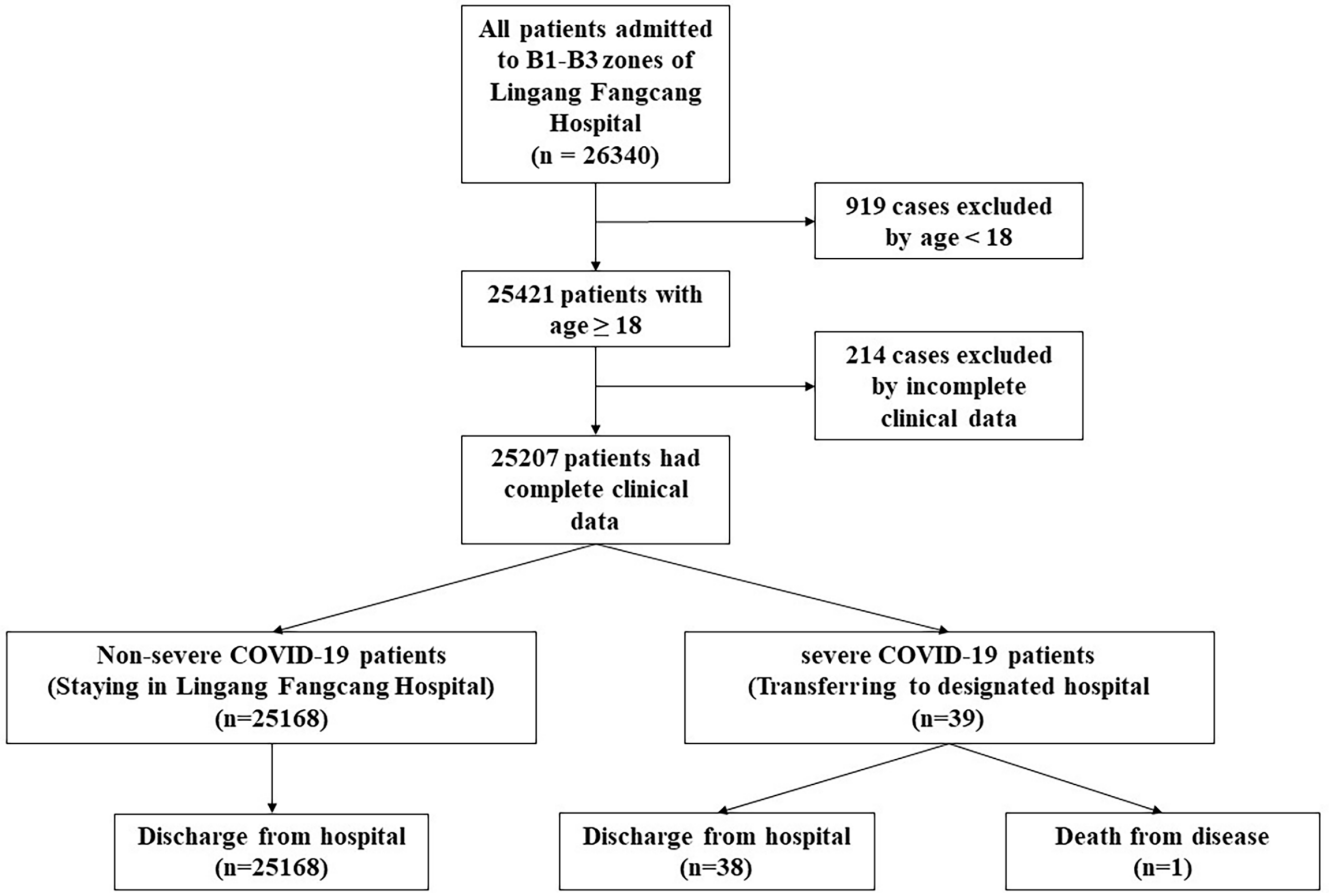
The flowchart of COVID-19 patients admitted to Lingang Fangcang hospital.

According to the criteria of inclusion and exclusion, a total of 25,207 patients with the omicron variant infection were enrolled in this study (**Figure 1**). Among them, there were 39 severe cases and 25,168 non-severe cases. Of the 39 severe cases who transferred to the designated hospital, there was one patient died for COVID-19. The other 38 severe cases were recovered and discharged from the designated hospital. Demographic and clinical features are summarized in **Table 2**. The median age was 45 years (IQR 33-54), and 65% patients were male. Cough (44.5%) and expectoration (38.4%) were the most two common symptoms. Hypertension (10.4%) and diabetes (3.5%) were most two common comorbidities. Most patients (81.1%) were fully vaccinated. The unvaccinated and partially vaccinated patients were 15.1% and 3.9%, respectively. The length of VST and hospital day were six days (IQR 4-9) and five days (IQR 4-7) in non-severe patients, respectively.

**Table 2:** Clinical characteristics of all COVID-19 patients infected by the omicron variant of SARS-CoV-2.

	All patients (n=25,207)	Severe patients (n=39)	Non-severe patients (n=25,168)	P value
**Gender, No. (%)**				0.313
Male	16395 (65)	22 (56.4)	16374 (65.1)	
Female	8812 (35)	17 (43.6)	8797 (34.9)	
**Age, median (IQR)**	45 (33,54)	59 (47,73)	45 (33,54)	<0.001
**Age≥65, No. (%)**	1952 (7.7)	17 (43.6)	1935 (7.7)	<0.001
**Syndromes, No. (%)**				
Fever	2027 (8)	8 (20.5)	2019 (8)	0.011
Cough	11210 (44.5)	11 (28.2)	11199 (44.5)	0.054
Expectoration	9678 (38.4)	10 (25.6)	9668 (38.4)	0.140
Fatigue	3192 (12.7)	12 (30.8)	3180 (12.6)	0.002
Nasal congestion	5918 (23.5)	4 (10.3)	5914 (23.5)	0.057
Runny nose	4903 (19.5)	3 (7.7)	4900 (19.5)	0.068
Sore throat	5848 (23.2)	6 (15.4)	5842 (23.2)	0.342
Body aches	2642 (10.5)	5 (12.8)	2637 (10.5)	0.598
Taste disorders	63 (0.2)	1 (2.6)	62 (0.2)	0.093
Diarrhea	264 (1)	1 (2.8)	263 (1)	0.337
**Comorbidities**				
Hypertension	2628 (10.4)	12 (30.8)	2616 (10.4)	<0.001
Diabetes	894 (3.5)	4 (10.3)	890 (3.5)	0.048
COPD/Asthma	119 (0.5)	2 (5.1)	117 (0.5)	0.015
Cancers	34 (0.1)	0 (0)	34 (0.1)	–
Others	227 (0.9)	2 (5.1)	225 (0.9)	0.048
**Vaccination acceptance history**				<0.001
None	3797 (15.1)	14 (35.9)	3783 (15)	
Partly vaccinated	977 (3.9)	3 (7.7)	974 (3.9)	
Fully vaccinated	20433 (81.1)	22 (56.4)	20411 (81.1)	
**Traditional Chinese Medicine**	11362 (45.1)	15 (38.5)	11347 (45.1)	0.503

Data are the median (interquartile range) or number of patients (percentage) unless otherwise indicated. Other comorbidities includes HBV, rheumatoid, chronic cholecystitis, Parkinson disease, epilepsy, hypothyroidism/hyperthyroidism, ankylosing spondylitis, gout, and chronic kidney disease.

### Risk factors for progression to severe COVID-19 (including death)

In order to evaluate the risk factors of deterioration among COVID-19 cases with omicron variant, we performed univariate and multivariate logistic regression analysis. The results of univariate logistic regression analysis showed that age, elderly(age > 65), some symptoms (fever, cough, fatigue and taste disorders), comorbidities (hypertension, diabetes and COPD/asthma) and vaccination history were independent risk factors for the deterioration to severe COVID-19 (including death) (**Table 3**). Then, multivariate logistic regression analysis suggested that age (OR=1.062, 95%CI 1.034-1.090, p<0.001), fever (OR=2.603, 95%CI 1.061-6.384, p=0.037), cough (OR=0.276, 95%CI 0.119-0.637, p=0.003), fatigue (OR=4.677, 95%CI 1.976-11.068, p<0.001), taste disorders (OR=14.917, 95%CI 1.884-118.095, p=0.010), and comorbidity (OR=2.134, 95%CI 1.059-4.302, p=0.034) were predictors for deterioration of COVID-19 (**Table 3**).

**Table 3:** Univariate and multivariate logistic regression analysis of risk factors for non-severe to severe COVID-19 (including death).

Items	Univariate analysis			Multivariate analysis		
	OR (95%CI)	P value	OR (95%CI)		P value
Age	1.078 (1.053-1.103)	<0.001	1.062 (1.034-1.090)		<0.001
Age>65	9.278 (4.919-17.501)	<0.001			
Female	1.439 (0.764-2.710)	0.261			
Fever	2.959 (1.358-6.445)	0.006	2.603 (1.061-6.384)		0.037
Cough	0.490 (0.244-0.985)	0.045	0.276 (0.119-0.637)		0.003
Expectoration	0.553 (0.269-1.135)	0.106			
Fatigue	3.073 (1.555-6.072)	0.001	4.677 (1.976-11.068)		<0.001
Nasal congestion	0.372 (0.132-1.047)	0.061			
Runny nose	0.345 (0.106-1.120)	0.076			
Sore throat	0.601 (0.252-1.436)	0.252			
Body aches	1.256 (0.491-3.215)	0.634			
Taste disorders	10.656 (1.440-78.831)	0.020	14.917 (1.884-118.095)		0.010
Diarrhea	2.492 (0.341-18.217)	0.368			
Number of symptoms (>3)	0.650 (0.272-1.553)	0.333			
Comorbidity	5.076 (2.693-9.569)	<0.001	2.134 (1.059-4.302)		0.034
Hypertension	3.831 (1.939-7.572)	<0.001			
Diabetes	3.118 (1.106-8.791)	0.032			
COPD/Asthma	11.574 (2.757-48.576)	0.001			
Vaccination acceptance history					
None	–	–			
Partly vaccinated	0.832 (0.239-2.902)	0.773	2.173 (0.586-8.060)		0.246
Fully vaccinated	0.291 (0.149-0.570)	<0.001	0.565 (0.275-1.163)		0.121

## Discussion

In late February of this year, an unexpected outbreak of SARS-CoV-2 infection rapidly arrived in Shanghai, China. In accordance with the data of Shanghai Municipal Health Commission, this COVID-19 ware was related to the omicron variant of SARS-CoV-2. The omicron variant is the recent mutation of SARS-CoV-2, which is reported 3.6-4.2 times higher effective reproduction number than the delta variant (Vaira et al., 2020; Nishiura et al., 2021). In this retrospective study, we evaluated 25,207 patients of the omicron variant infection from Lingang Fangcang Hospital, the third largest Fangcang hospital in Shanghai. All the patients were linked to this 2022 outbreak of COVID-19 in Shanghai. The main finding was that age, fever, cough, fatigue, taste disorders, fatigue, and comorbidity were predictors for deterioration of COVID-19 with the omicron variant of SARS-CoV-2. To the best of our knowledge, there is still a lack of a large-scale study to assess the clinical characteristics and risk factors for disease progression with SARS-CoV-2 Omicron variant infection.

Among 25,207 patients, there were more male patients (65%) than females (35%). Cough (44.5%) and expectoration (38.4%) were the most two common symptoms. Hypertension (10.4%) and diabetes (3.5%) were most two common comorbidities. These are similar to other Fangcang studies reported before (Cheng et al., 2020; Gu et al., 2021; Mueller et al., 2022). Fangcang hospital represents a novel concept of public health, which is to transform public venues to large and temporary hospitals to isolate patients with non-severe COVID-19 patients (Lv and Lv, 2021). In this wave of omicron variant in Shanghai, most of patients were non-severe cases. Fangcang hospitals in Shanghai isolated tens of thousands of patients and provided high-quality medical care. However, there are still some COVID-19 patients who under risk to severe or even critical condition. Our results showed that 39(0.15%) of 25207 patients were deteriorated and transferred to the designated hospital, which was much less than transfer rate of Fangcang hospitals reported in other COVID-19 waves (Wang et al., 2020; Venturini et al., 2021; Liu et al., 2021). This result indicated that COVID-19 patients of the omicron variant infection presented a lessened clinical severity and degraded virulence in comparison of the wild-type strain or other VOCs. Our results showed that most of cases (81.1%) were fully vaccinated, which might contribute to the low clinical severity rate of this Shanghai omicron wave. These results accent the importance of timely vaccination.

Our results showed that age, fever, cough, fatigue, taste disorders, fatigue, and comorbidity were predictors for deterioration of the omicron variant infection. Our results demonstrated that severe patients were older than non-severe patients. The number of elderly cases in severe group was larger than that of non-severe group. Both univariate and multivariate analysis indicated that age was the independent risk for non-severe to severe COVID-19. This finding is compatible with previous studies (Chen et al., 2020; Song et al., 2020; Yan et al., 2020). Elderly patients have poorer immunity condition and more comorbidities than younger patients, which are the disadvantages of recovery and rehabilitation. Thus, as to elderly COVID-19 patients, it requires more attention and earlier intervention to prevent the deterioration or even death.

It is widely recognized that COVID-19 patients with chronic disease are under higher risk to progress into critical condition or even death. Our results indicated that severe cases had a significantly higher frequency of comorbidities than that of other cases. Univariate logistic regression analysis showed that the history of hypertension, diabetes or COPD/asthma, or preexisting comorbid conditions are risk factors for the deterioration to severe COVID-19 (including death). However, after adjusting the confounders, including age, sex, symptoms and vaccination, we found that only history of comorbidity presented to be the factor of COVID-19 deterioration. COVID-19 cases with hypertension, diabetes or COPD are more likely to get worse or even death (Lippi and Plebani, 2020; Onder et al., 2020). These chronic diseases may induce the changes of Angiotensin-converting enzyme 2 expression and enhance the lung injury of SARS-CoV infection (Letko et al., 2020).

Some studies on large sample of Fangcang hospital cases showed that the most common symptom of COVID-19 was fever (Wang et al., 2020; Lv and Lv, 2021; Liu et al., 2021). In our study of 25,207 patients, cough (44.5%) and expectoration (38.4%) were the most two common symptoms. 8% of patients suffered fever, which was lower than the numbers reported before (Wang et al., 2020; Lv and Lv, 2021; Liu et al., 2021). This may contribute to the reduced virulence of the omicron variant and the high vaccination rates. Our results showed that fever or fatigue were more common in severe group than those in non-severe group. It has been proved that infection of SARS-CoV can cause the disorder of immune response, which triggers a deleterious storm of inflammation (Ye et al., 2020; Soy et al., 2021). It was reported that persistent fever more than seven days after onset could suggest a higher risk of poor prognosis in COVID-19 patients, whereas short-time defervescence indicated a better outcome in spite of the thermal spike (Ng et al., 2020). No matter the period of infection and rehabilitation of COVID-19, fatigue is one of the most relevant symptoms. Fatigue manifests as a sensation ranging from tiredness to exhaustion, which is a very complex physical and triggered by a serial of pathological mechanisms (Azzolino and Cesari, 2022). As reported, 45% of the COVID-19 patients demanded a decreased work intensity in comparison to pre-infection, and 22.3% resigned at 7 months follow up due to fatigue (Bansal et al., 2022). Taken together, it is meaningful to perform further investigation on the clinical value of fever pattern and mechanism of fatigue in the infection of omicron variant of SARS-CoV-2. Smell and taste dysfunction were consistently reported among the most common symptoms of COVID‐19 with about 65%–70% of mild‐to‐moderate cases experiencing a chemosensory impairment during the acute phase of the COVID‐19 (Spinato et al., 2020). Our results indicated that only 0.2% cases suffered from taste disorders. A prospective study demonstrated that the prevalence and the severity of COVID‐19–associated smell and taste dysfunction had dropped significantly with the advent of the Omicron variant but it still remained above 30% (Boscolo-Rizzo et al., 2022). The low prevalence of taste disorders in our study may due to the deficiency of systematic assessment about chemosensory function. Further thorough and professional research is needed to assess the chemosensory dysfunction in the Omicron variant patients.

Despite the large sample size, there are some limitations in this study. First of all, the information about SARS-CoV-2 strain was unavailable for individual patients. However, as described in the Methods section, the omicron was the primary strain in this Shanghai COVID-19 wave. Therefore, we believe that our patients are largely representative of patients with omicron variant. Secondly, considering it’s a retrospective study, a prospective study is needed to validate our conclusion. Thirdly, by reason of the facility limitations in Fangcang hospital, routine blood and markers test of hepatic and renal functional were not deficiency in most patients during their hospitalization. There are few severe COVID-19 patients in this 2022 Shanghai COVID-19 epidemic. There were only 39 patients in severe group and 1 patient dead. This study lacked data on novel drug treatment, such as nirmatrelvir/ritonavir. lastly, some data were deficiency, including information on vaccine types and body mass index. We used online questionnaire for the collection of data. COPD and asthma were grouped in one item in the questionnaire.

In conclusion, our study of 25,207 cases indicates that deteriorated patients have different clinical features compared with non-deteriorated patients in this 2022 Shanghai. Age, fever, cough, fatigue, taste disorders, and comorbidity are predictors for deterioration of the omicron variant infection.

## Data availability statement

The raw data supporting the conclusions of this article will be made available by the authors, without undue reservation.

## Ethics statement

The studies involving human participants were reviewed and approved by Clinical Research Ethics Committee of Jiangsu Province Hospital of Chinese Medicine. Written informed consent for participation was not required for this study in accordance with the national legislation and the institutional requirements. Written informed consent was not obtained from the individual(s) for the publication of any potentially identifiable images or data included in this article.

## Author contributions

JH conceived the research. PY-H, GY-Y, ZH-D, CQ-H, GX-R and ZH-Q collected and analyzed the clinical data. JH designed the study. PY-H drafted the manuscript. JH reviewed the final manuscript. All authors read and approved the final manuscript.

## Funding

This work was supported by the National Natural Science Foundation of China under Grant (81700243), the subject of Jiangsu Province Hospital of Chinese Medicine under Grant (Y2020CX42), and the subject of National Base for Disease Prevention and Control of Chinese Medicine.

## Acknowledgments

The authors would like to thank all of the participants for their time and effort.

## Conflict of interest

The authors declare that the research was conducted in the absence of any commercial or financial relationships that could be construed as a potential conflict of interest.

## Publisher’s note

All claims expressed in this article are solely those of the authors and do not necessarily represent those of their affiliated organizations, or those of the publisher, the editors and the reviewers. Any product that may be evaluated in this article, or claim that may be made by its manufacturer, is not guaranteed or endorsed by the publisher.

## Abbreviations

SARS-CoV-2, Severe acute respiratory syndrome coronavirus 2; COVID-19, Coronavirus disease 2019; VOCs, variants of concerns; VST, viral shedding time.
